# Biallelic inheritance in a single Pakistani family with intellectual disability implicates new candidate gene *RDH14*

**DOI:** 10.1038/s41598-021-02599-z

**Published:** 2021-11-30

**Authors:** Stephen F. Pastore, Tahir Muhammad, Ricardo Harripaul, Rebecca Lau, Muhammad Tariq Masood Khan, Muhammad Ismail Khan, Omar Islam, Changsoo Kang, Muhammad Ayub, Musharraf Jelani, John B. Vincent

**Affiliations:** 1 Molecular Neuropsychiatry and Development (MiND) Lab, Campbell Family Mental Health Research Institute, Centre for Addiction and Mental Health, 250 College Street, Toronto, ON M5T 1R8 Canada; 2 Institute of Medical Science, University of Toronto, Toronto, ON Canada; 3 Department of Pathology, North-West School of Medicine, Hayatabad, Peshawar, Khyber Pakhtunkhwa Pakistan; 4 Department of Zoology, Islamia College Peshawar, Peshawar, Khyber Pakhtunkhwa Pakistan; 5 Department of Diagnostic Radiology, Queen’s University, Kingston, ON Canada; 6 Department of Biology and Institute of Basic Sciences, Sungshin Women’s University, Seoul, Republic of Korea; 7 Department of Psychiatry, Queen’s University Kingston, Kingston, ON Canada; 8 Centre for Omic Sciences, Islamia College Peshawar, Peshawar, Khyber Pakhtunkhwa Pakistan; 9 Department of Psychiatry, University of Toronto, Toronto, ON Canada

**Keywords:** Neurological disorders, Movement disorders, Neurodevelopmental disorders

## Abstract

In a multi-branch family from Pakistan, individuals presenting with palmoplantar keratoderma segregate in autosomal dominant fashion, and individuals with intellectual disability (ID) segregate in apparent autosomal recessive fashion. Initial attempts to identify the ID locus using homozygosity-by-descent (HBD) mapping were unsuccessful. However, following an assumption of locus heterogeneity, a reiterative HBD approach in concert with whole exome sequencing (WES) was employed. We identified a known disease-linked mutation in the polymicrogyria gene, *ADGRG1*, in two affected members. In the remaining two (living) affected members, HBD mapping cross-referenced with WES data identified a single biallelic frameshifting variant in the gene encoding retinol dehydrogenase 14 (*RDH14*). Transcription data indicate that *RDH14* is expressed in brain, but not in retina. Magnetic resonance imaging for the individuals with this *RDH14* mutation show no signs of polymicrogyria, however cerebellar atrophy was a notable feature. RDH14 in HEK293 cells localized mainly in the nucleoplasm. Co-immunoprecipitation studies confirmed binding to the proton-activated chloride channel 1 (PACC1/TMEM206), which is greatly diminished by the mutation. Our studies suggest *RDH14* as a candidate for autosomal recessive ID and cerebellar atrophy, implicating either disrupted retinoic acid signaling, or, through PACC1, disrupted chloride ion homeostasis in the brain as a putative disease mechanism.

## Introduction

**Figure 1:**
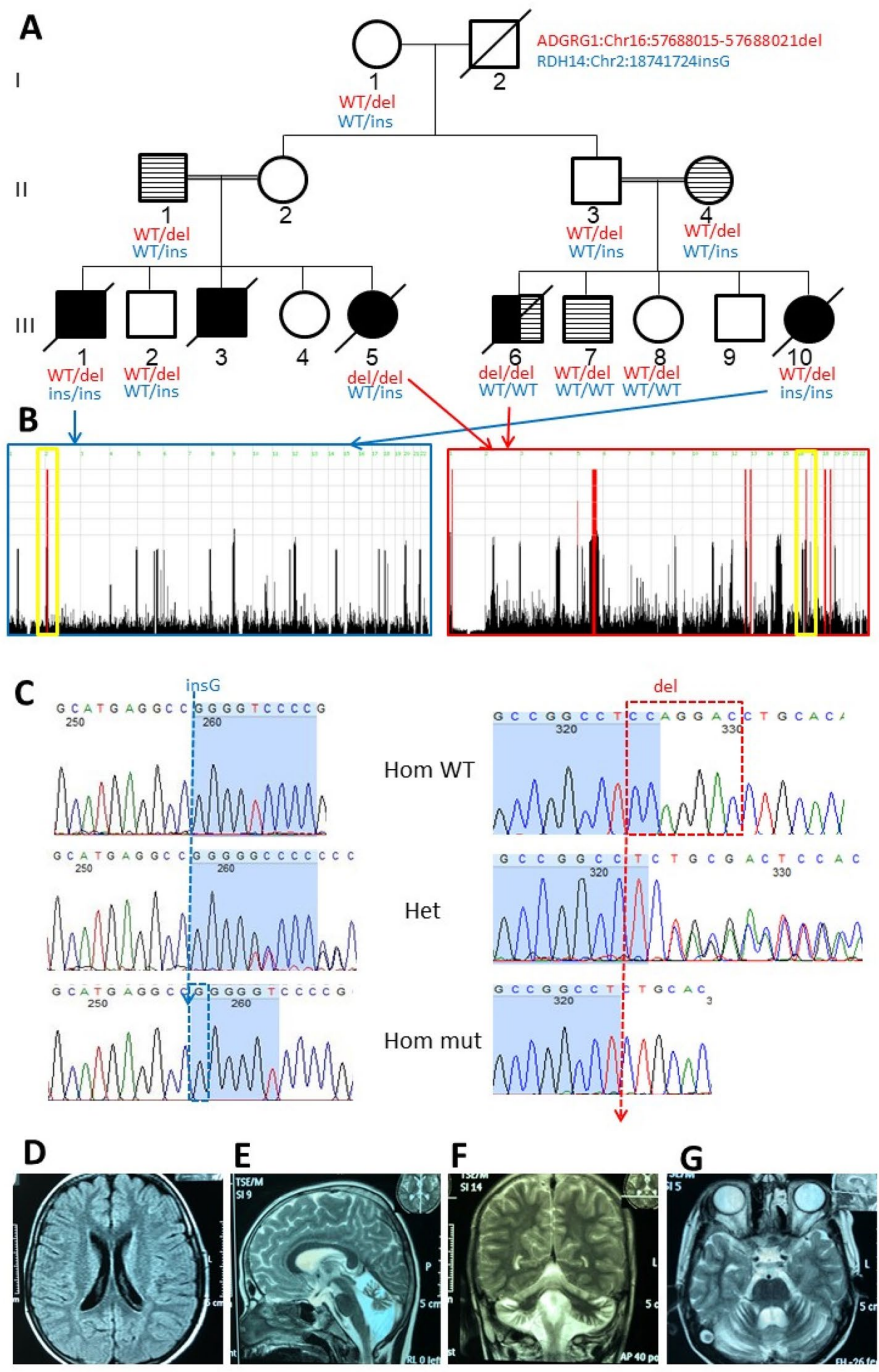
Homozygosity mapping and *ADGRG1*/*RDH14* mutations in Pakistani intellectual disability/hyperkeratosis family. (**A**) Pedigree drawing focusing on branches with ID. Additional branches with individuals with hyperkeratosis have been trimmed. Individuals II-1 and II-4 are siblings, whose mother was sibling of I-1, and are first cousins of II-2 and II-3. Dark shaded shapes represent a diagnosis of ID, and hashed shading indicates autosomal dominant hyperkeratosis. III-6 has both diagnoses; (**B**) Homozygosity Mapper^23^ output for Illumina Human CoreExome genotypes, left, using III-1 and III-10 as cases, and III-2, III-5, III-6, III-7, III-8 as controls, and right, using III-5 and III-6 as cases, and III-1 and III-11, III-2, III-7, III-8, and III-10 as controls. HBD regions are listed in Table 1. Whole exome sequencing (WES) identified a known disease-causing mutation in the gene for bilateral frontoparietal (BFPP) gene *ADGRG1* (also known as *GPR56*), within the HBD region on chromosome 16 (indicated in a yellow box), and segregating just for individuals III-5 and III-6. WES also identified loss of function mutations in *RDH14*, within the sole HBD region for the remaining affecteds, III-1 and III-10. (**C**) Representative electropherograms for RDH14 (left) exon 1, and *ADGRG1* exon 5, for homozygous wild type (hom WT), heterozygous (het) and homozygous mutant (hom mut) genotypes. (**D**–**G**) Magnetic resonance imaging of RDH14 mutation homozygote individual III:1: (**D**) Axial FLAIR image showing normal cerebral white matter; (**E**) Sagittal T2 image showing marked volume loss of the cerebellar vermis, with associated ex-vacuo dilatation of the 4th ventricle. (**F**) Coronal T2 image showing severe cerebellar atrophy, including the lateral cerebellar hemisphere. Note preserved cerebral volume. (**G**) Axial T2 image showing marked volume loss of the cerebellum, with associated ex-vacuo dilatation of the 4th ventricle. Note preserved brainstem volume.

Frequently, studies of intellectual disability (ID) in multiplex consanguineous families can, through a process of mapping homozygosity-by-descent (HBD) regions and whole exome sequencing (WES), identify either disease-causing genes/mutations or likely candidates. Recent studies of large family cohorts, including our own, suggest the success rate is around 50% of families^1–5^, and this is anticipated to increase as additional studies either corroborate existing candidate genes or identify new candidates. However, that still leaves a significant proportion of families for which no clear candidate can be identified. In a proportion of the families, part of the problem lies in the high degree of genetic heterogeneity for intellectual disability, and larger pedigrees with multiple affected individuals in multiple branches are more likely to be prone to within-family heterogeneity. However, where HBD mapping and WES data for multiple family members is available, careful analysis and accommodation of potential heterogeneity can still be fruitful and even identify new candidate genes. Here, we identified a large consanguineous Pakistani family presenting with an apparently autosomal dominant form of palmoplantar keratoderma (PPK) across multiple branches, among which, in addition, there are five members with apparently autosomal recessive ID, one of whom died prior to the study, and four who have died since (Fig. 1A). Previously, through WES, a mutation in the *COL20A1* gene was identified and reported as a candidate cause of the PPK^6^. Through homozygosity-by-descent mapping and whole exome sequencing, and using an iterative analytic process, we identified two individuals from separate affected branches with a known mutation in the polymicrogyria gene, *ADGRG1*, and an early truncating mutation in a new candidate gene, retinol dehydrogenase 14 (*RDH14*), in the two remaining ID members.

## Results

### HBD mapping and exome sequence analysis

Autozygosity analysis of microarray genotype data using HomozygosityMapper was unable to identify a single homozygosity-by-descent (HBD) region for all four available affected ID individuals. Thus, in order to explore possible within-family genetic heterogeneity, a reiterative analysis was performed using HomozygosityMapper for subsets of three or two affecteds, with the other affecteds re-classified as controls purely for the purpose of analysis. Results are listed in Table 1, and HomozygosityMapper output shown in Fig. 1B.

**Table 1:** Homozygosity-by-descent (HBD) mapping using Illumina Human CoreExome genotype data for family members III-1, III-5, III-6, III-10 (affected), and III-2, III-7 and III-8 (unaffected), using Homozygosity Mapper^23^. As no HBD regions were identified for all four affecteds, in order to explore possible genetic heterogeneity, a reiterative analysis was performed for subsets of three or two affecteds, with the other affecteds re-classified as controls purely for the purpose of analysis. Coordinates provided in hg19.

HBD#	Chr	Start SNP	End SNP	Start bp	End bp	Size (Mb)	HM Score
	All affecteds: No HBD						
	III-1, III-5, III-6						
1	5	rs1508879	rs2522051	115,402,108	131,797,578	16.395	756
2	5	rs319594	rs12188192	134,250,146	136,352,840	2.103	750
	III-1, III-5, III-10: No HBD						
	III-5, III-6, III-10: No HBD						
	III-1, III-6, III-10: No HBD						
	III-5, III-6						
3	5	rs4607330	rs1508879	105,007,215	115,402,108	10.395	500
4	12	rs7134132	rs9989044	91,296,986	98,518,185	7.221	500
5	12	rs12578694	rs11147281	129,625,001	133,757,954	4.125	500
6	16	rs4784474	rs4404061	55,096,404	59,010,501	3.914	500
7	18	rs658513	rs9966082	10,100,136	20,132,564	10.032	500
	III-1, III-10						
8	2	rs818148	rs1473886	10,637,325	20,368,519	9.731	500
9	1	rs4600084	rs9436636	56,844,621	61,812,773	4.968	258
10	4	rs7679973	rs2375799	184,569,516	187,414,115	2.845	263
11	4	rs4077958	rs6827843	8,647,233	10,688,637	2.041	150
12	14	rs11157849	rs7142470	52,242,196	56,262,409	4.02	257
	III-1, III-5						
13	5	rs11745587	rs4345357	131,796,922	134,253,500	2.457	500
	III-1, III-6						
14	5	rs17521284	rs2906057	136,345,401	141,590,358	5.245	500
	III-5, III-10: no HBD						
	III-6, III-10: no HBD						

Using a custom in-house analytic pipeline, a known disease-associated homozygous variant was identified within HBD region #6 (Table 1; individuals III-5 and III-6, Fig. 1A), within the adhesion G-protein-coupled receptor G1 (*ADGRG1*; MIM 604110) gene. Mutations in *ADGRG1*, also known as *GPR56,* are known to cause autosomal recessive bilateral frontoparietal polymicrogyria (BFPP; MIM 606854), which includes intellectual disability as a main clinical feature. Sanger sequencing was used to confirm the variant, Chr16:57,688,015-57688021del; NM_005682.5:c.738del; p.(Gln247Argfs*76) (rs587776625), and to check segregation across the family (electropherograph shown in Fig. 1C). The genotypes for this variant are provided with the pedigree drawing, Fig. 1A, where it is apparent that, as predicted by the HBD analysis (see HBD#6 in Table 1), the variant is in both branches but only segregating with one affected individual in each branch (III-5 and III-6), with the remaining two affected individuals (III-1 and III-10) as heterozygous carriers. This 7 bp deletion variant was initially reported in a family with BFPP from the Gujarat region of India, as well as in families from Pakistan and Afghanistan, suggesting a founder mutation stably maintained within a large population^7^. Thus, we were confident in our assertion that this was the disease-causing mutation in these two family members, and excluding them from further analysis. Brain magnetic resonance imaging (MRI) was not available for these two individuals.

The analysis for the two remaining affecteds, III-1 and III-10, indicated five regions of significant HBD (Table 1, HBD #s 8 to 12). WES analysis for individual III-1, restricted to these HBD regions, revealed just a single rare (MAF < 0.001) homozygous variant predicted as damaging- a 1 bp insertion variant: Chr2:18741725dup; NM_020905.3:c.114dup; Gly39Argfs*97. This variant is located in exon 1 of a two-exon gene, retinol dehydrogenase 14 (*RDH14*; MIM 616796), and is predicted to lead to an early protein truncation as well as nonsense-mediated mRNA decay (according to mutationtaster.org). Sanger sequencing was used to confirm the variant, and to check segregation within the family (genotypes shown in Fig. 1A, electropherographs shown in Fig. 1C). The genotypes for this variant are provided with the pedigree drawing, Fig. 1A, and support the HBD analysis restricted to affecteds III-1 and III-10 (see HBD#6 in Table 1). This variant is present in the gnomAD v2.1.1 control exome dataset (non-neurological diagnosis subset; gnomad.broadinstitute.org) at a frequency of 9.7 × 10^–5^ (11 alleles out of 113,436), and with no homozygotes present. The frequency is slightly higher within a subset of alleles from South Asian controls (MAF = 2.5 × 10^–4^). Also of note, there were no loss-of-function variants in *RDH14* in the gnomAD dataset, except at very low frequency (MAF < 0.001), and no homozygotes. Analysis of X-chromosomal WES data for rare hemizygous damaging variants in affected males found nothing.

### Brain magnetic resonance imaging (MRI) and clinical information

Brain MRI through T1/T2 weighted images and T1-weighted post-contrast images for III-1 (affected male at age 14 years) and III-10 (affected female at age 20 months) showed no signs of polymicrogyria. Analysis of MRI images for III-1 indicated significant loss of volume affecting the cerebellum and the cerebellar vermis in particular (Fig. 1D–G). There is volume loss but to a lesser degree affecting the lateral cerebellar hemispheres, and, in the setting of a normal-sized posterior fossa, a post-natal etiology for volume loss is suggested. In female individual III-10 (at 20 months of age), mild cerebellar atrophy (and the vermis in particular) was evident. Although full neurological examination has not been possible, it has been reported that both individuals III-1 and III-10 were able to toddle until about 3 years of age, but never progressed to walking, and, having lost all mobility, have been wheelchair-bound ever since, which suggests the involvement of a severe form of movement disorder in addition to intellectual disability.

### Protein molecular/functional studies

**Figure 2:**
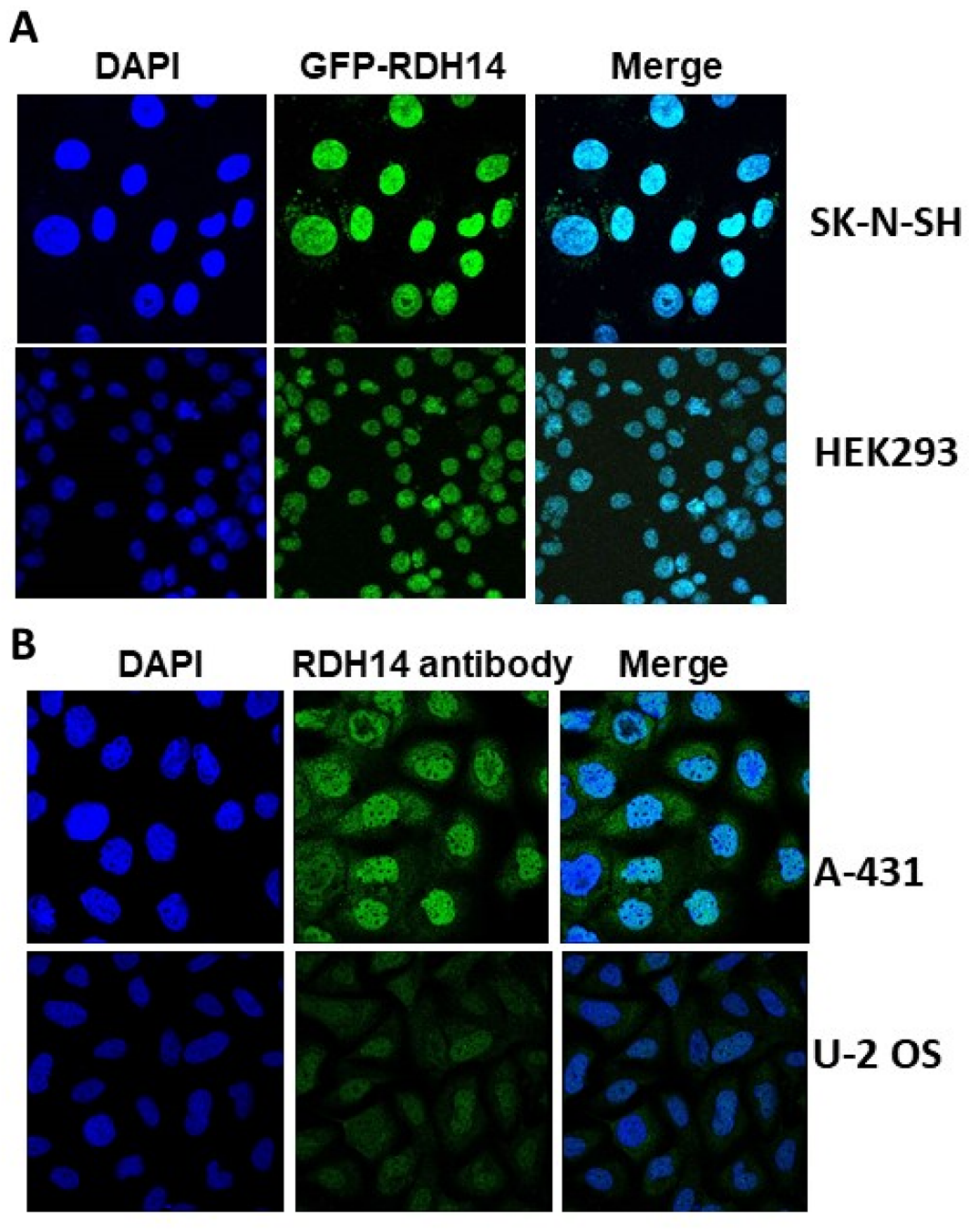
Cellular localization of RDH14. (**A**) using SK-NS-H cells (neuroblastoma) and HEK293T cells, transfected with RDH14-GFP in a pDEST53 construct. (**B**) Images from http://proteinatlas.org^28^, shared under a Creative Commons Attribution License. RDH14 polyclonal antibody HPA056686 (MilliporeSigma; St Louis, MO); Human assay: A-431 fixed with PFA, dilution: 1:49; Human assay: U-2 OS fixed with PFA, dilution: 1:49.

**Figure 3:**
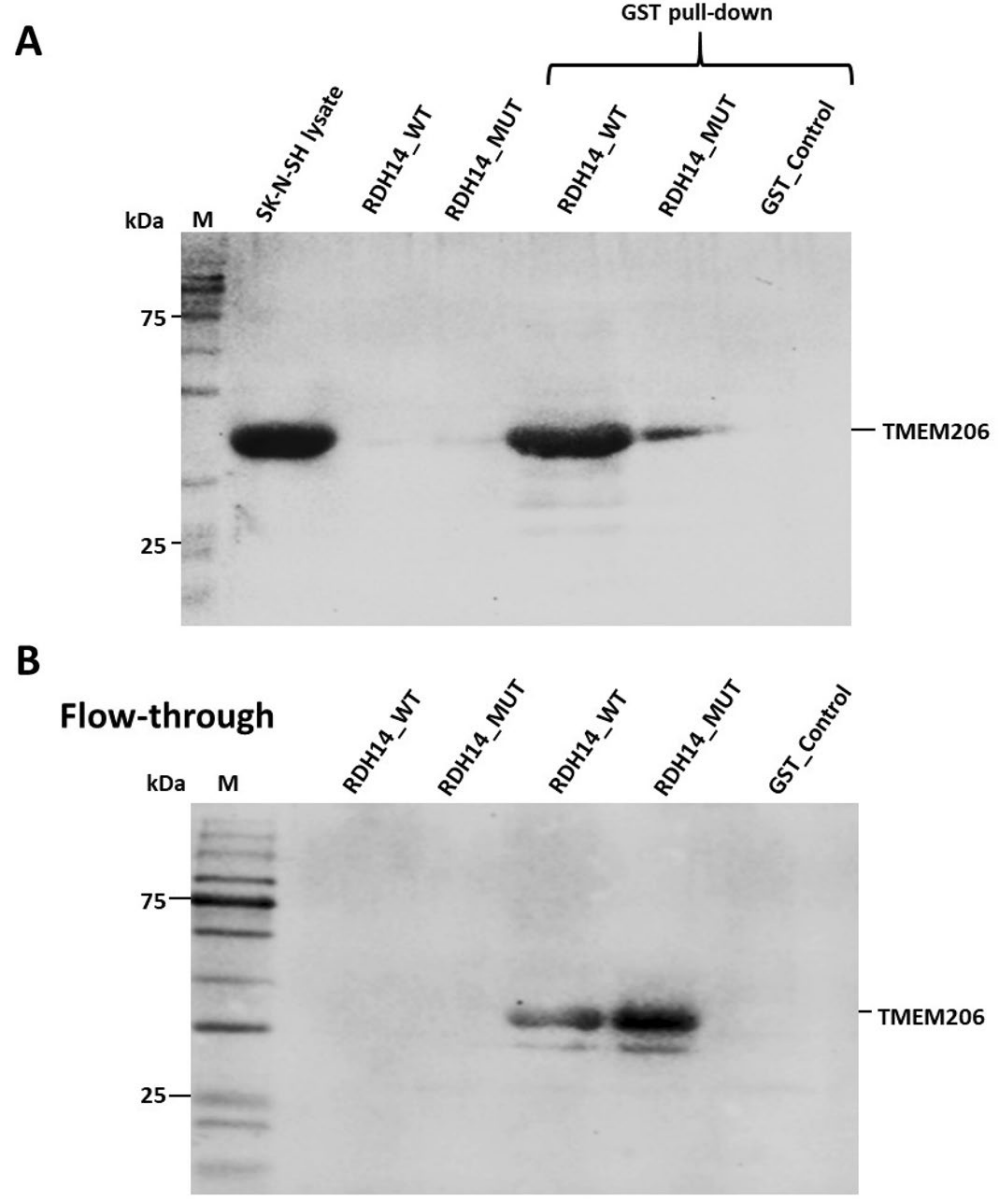
Western blotting showing GST pull-down of TMEM206/PACC1 from SK-N-SH cell lysate using wild-type (WT) versus mutant RDH14-GST constructs. A TMEM206 (PACC1) rabbit polyclonal antibody (1:1000 dilution; catalogue #TA342366; Origene Technologies, Rockville, MD) was used. (**A**) Input to lane 1 was SK-N-SH cell lysate (as a positive control). Lanes 2 and 3 show extracted protein for the RDH14-GST constructs, WT and mutant, respectively (as negative controls). In lanes 4, 5, and 6, the GST pull-down eluted protein, for WT and mutant RDH14-GST, and GST alone (as negative control), respectively (i.e. bait protein against prey input). (**B**) Flow-through from lanes 2–6 above was run and probed with the TMEM206 antibody. Western hybridization using GST antibodies was also performed to check sample loading (Supplementary Fig. S2).

Over-expression of an RDH14-GFP construct in HEK293 cells and SK-N-SH neuroblastoma (undifferentiated) cells suggest that RDH14 protein expression in cells is chiefly in the nucleoplasm (Fig. 2). The BioGRID database (thebiogrid.org) indicated evidence of protein–protein interaction between RDH14 and the TMEM206 protein, also known as proton-activated chloride channel 1 (PACC1), using affinity capture-mass spectrometry^8^. We demonstrated that an RDH14-GST wild type construct successfully pulled down PACC1 (Fig. 3). An RDH14-GST construct with a truncating mutation mimicking that identified in ID individuals showed the ability to interact with PACC1 was greatly reduced, and no pull-down was seen for a GST control (Fig. 3). BioGrid also identifies bone morphogenic protein/retinoic acid inducible neural-specific protein 2, BRINP2 (also known as FAM5B), as a putative interactor for RDH14. However GST-pull down was unable to confirm the interaction with RDH14.

**Figure 4:**
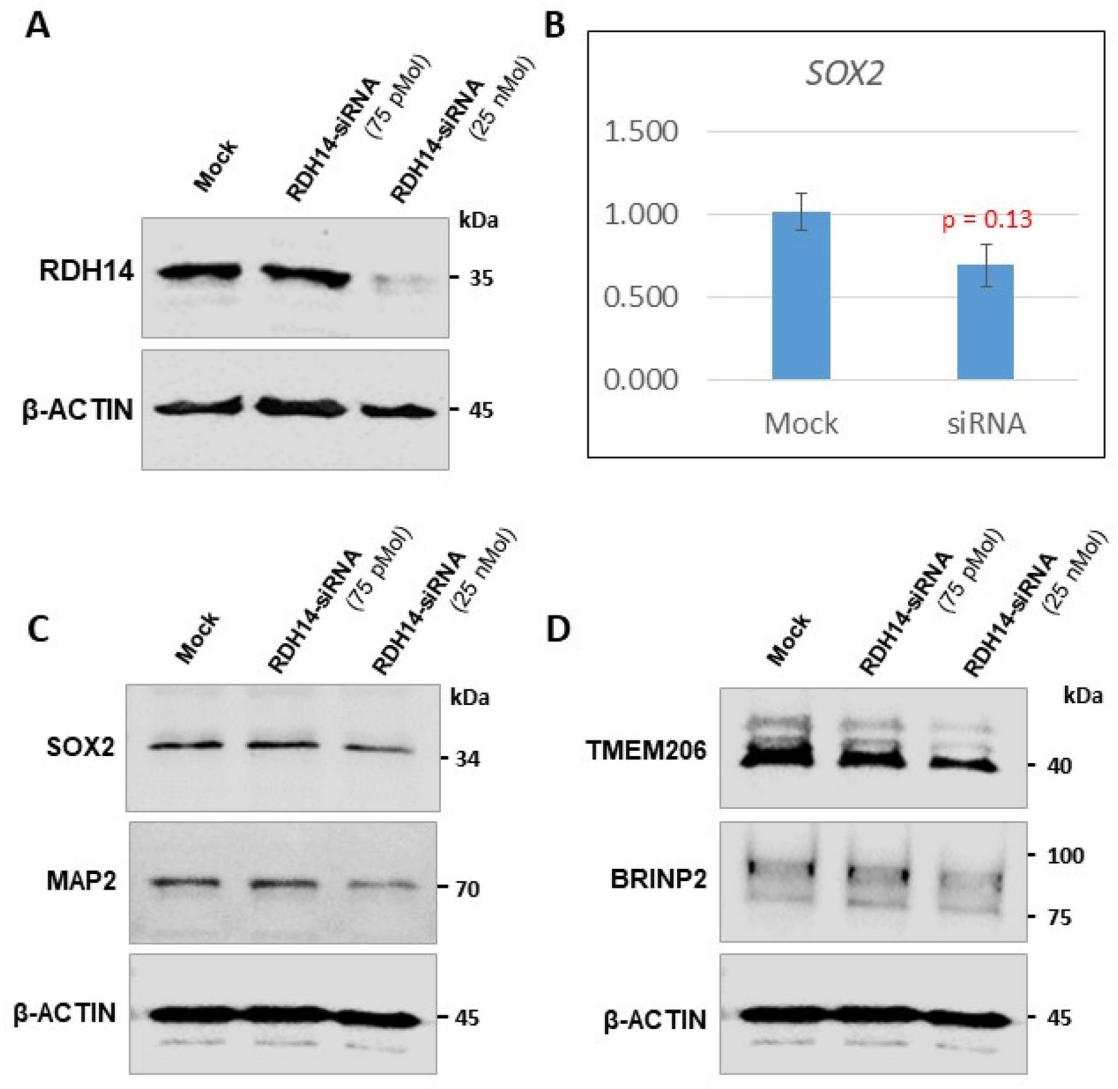
Analysis of the *RDH14* knock down effects on SOX2, MAP2, TMEM206 (PACC1) and BRINP2 (FAM5B) in SK-N-SH cells. (**A**) Western blot analysis of the RDH14-siRNA transfection showing knock down of RDH14 protein, using anti-RDH14 rabbit polyclonal antibody (1:1000 dilution; cat# HPA056686; Atlas Antibodies, Bromma, Sweden). (**B**) qRT-PCR showing effect of RDH14-siRNA on transcription of SOX2 in SK-N-SH cells. (**C**) Representative western blot images showing the effects of RDH14 siRNA knock down on SOX2 and MAP2 protein levels using anti-SOX2 monoclonal antibody (1:2000 dilution; cat# ab79351, Abcam Inc, Toronto, ON), and anti-MAP2 polyclonal antibody (1:2000 dilution; cat# ab79351, Abcam Inc, Toronto, ON). (**D**) Effect of RDH14 siRNA knock down on TMEM206/PACC1 and FAM5B/BRINP2 protein levels using anti-TMEM206 rabbit polyclonal antibody (1:1000 dilution; cat# TA342366; Origene Tech., Rockville, MD), and anti-FAM5B rabbit polyclonal antibody (1:500 dilution; cat# orb474203; Biorbyt Tech., St Louis, MO).

Although analysis of the effects of knocking out or knocking down *RDH14* on neuronal differentiation and maturation was not feasible, as it is difficult to predict the effects of lack of retinol dehydrogenase 14 enzyme when utilizing retinoic acid as the standard differentiation agent on neuroblastoma cell lines, we were able to effectively knock-down expression of endogenous *RDH14* in SK-N-SH cells. Western analysis of well-studied factors associated with cell pluripotency and dendritic marker (SOX2 and MAP2, respectively) and putative RDH14 interactors (BPRIN2, TMEM206, also MAP2), show a trend towards decreased expression in the *RDH14*-silenced cells for SOX2, MAP2 and TMEM206 (Fig. 4). RT-qPCR also showed reduced transcription for *SOX2* in *RDH14*-knockdown cells, although non- significant (Fig. 4). RT-qPCR was also performed on mock versus *RDH14* silenced cells for neuronal differentiation factor genes *OCT4*, *RAR-A*, *NEUROD1*, *NESTIN*, and *KI67*, but no change in transcription levels was observed (data not shown; raw data is available from the corresponding author upon request).

## Discussion

*RDH14* encodes a member of the SDR7C family short-chain dehydrogenase reductase enzymes, which, in addition to RDH14 (SDR7C4), includes RDH11 (SDR7C1), RDH12 (SDR7C2) and RDH13 (SDR7C3) (see^9^ for review). SDR7C enzymes are involved in retinoic acid catabolism and homeostasis, catalyzing the conversion of retinaldehyde to retinol, as well as the reverse, conversion of retinol to retinaldehyde^10^. Retinaldehyde is converted by aldehyde dehydrogenases to retinoic acid (RA). All-trans RA is a critical developmental morphogen, important for the fine control of differentiation and cell-patterning. RA is critical to embryonic developmental structures, including forebrain and hindbrain, but also likely to continue to function throughout adulthood (reviewed by^11^). The RDH14 protein is reported to be ubiquitously expressed, including brain, but notably not in the eye, whereas other SDR7C members are expressed in specific non-CNS tissues (RDH11: prostate, testis, kidney; RDH13: retina, endocrine tissue, lung, gastrointestinal tract, liver, pancreas, kidney, skin, male and female-specific tissues; http://proteinatlas.org; N.B. RDH12 protein data is unavailable, but transcription is highest in skin). RNA in situ hybridization studies of *RDH12* show localization to the base of photoreceptor inner segments^12^. Biallelic mutations in *RDH12* cause Leber congenital amaurosis-13 (LCA13; MIM 612712)^13^. Biallelic truncating mutations in *RDH11* cause a form of retinitis pigmentosa (RP) with delayed psychomotor development, learning difficulties, and distinct craniofacial and physical dysmorphologies (MIM 616108^14^). RDH14 exhibits retinaldehyde reductase activity, with preference for *all-trans-*retinaldehyde as a substrate over *9-cis*-retinaldehyde, with higher catalytic efficiency compared to RDH11, and with reductase activity albeit at a lower level^10^. The variant reported here would leave the single putative transmembrane domain intact, but would remove the co-factor binding domain at Gly50, and the entire SDR enzymatic domain including the putative active site at Tyr217.

Although reports indicate RDH14 is expressed in microsomes^10^, our own data using over-expression of an RDH14-GFP construct (in HEK293 cells and SK-N-SH neuroblastoma cells, as well as data from The Human Protein Atlas (http://www.proteinatlas.org) using antibodies against endogenous RDH14 in A-431 (squamous carcinoma) and U-2-OS (osteosarcoma) cells, suggest that RDH14 protein is also strongly expressed in the nucleoplasm (Fig. 2). This is somewhat surprising, as, although RA has nuclear action, it is assumed that its synthesis is cytoplasmic. Also, no nuclear localization signal could be identified (using SeqNLS (http://mleg.cse.sc.edu/seqNLS/), or NLStradamus (http://www.moseslab.csb.utoronto.ca/NLStradamus), NLS Mapper (http://nls-mapper.iab.keio.ac.jp/cgi-bin/NLS_Mapper_form.cgi), NucPred (https://nucpred.bioinfo.se/nucpred/). Moreover localization predictions suggest RDH14 to by overwhelmingly cytoplasmic, and with no predicted DNA or RNA binding motifs (https://psort.hgc.jp). Interestingly, another retinol dehydrogenase, RDH8 (all-*trans*), is also shown by immunocytochemistry to be nucleoplasmic (The Human Protein Atlas; http://www.proteinatlas.org).

We explored the validity of several reported protein–protein interactions for RDH14 as reported through http://thebiogrid.org. We confirmed experimentally through GST-pull-down, using a RDH14-N-terminal GST construct that RDH14 binds with proton-activated chloride channel 1 (PACC1, previously TMEM206), as reported through http://thebiogrid.org, using affinity capture-mass spectrometry^15^. GST-pull-down using an RDH14 construct with an early truncating mutation showed greatly reduced binding to PACC1 (Fig. 3). *PACC1*, which is transcribed at very high levels in the brain, is reported to form an acid-sensitive outwardly rectifying (ASOR) anion channel^16^. Disruption of PACC1 protects against acid-induced neuronal apoptosis and axonal degeneration^16^. The putative interaction with BRINP2 was not confirmed, however. The interaction between RDH14 and PACC1 is corroborated by single-cell RNAseq data from developing and adolescent mice that supports co-expression of the two genes (http://www.mousebrain.org^17^) in many brain cell types, particularly strongly in noradrenergic and cholinergic neuronal cells in sympathetic system. We speculate that RDH14, which possesses a single transmembrane domain, may have roles in addition to, and possibly independent of, its enzymatic function, however further work is required.

Interestingly, disruption of retinoic acid signaling in the brain is linked with a variety of common intellectual disability disorders. For instance, one of the most common missense mutations in the Rett syndrome gene, *MECP2*, disrupts binding at the nuclear receptor corepressor (NCoR)/silencing mediator of retinoic acid and thyroid receptors (SMRT) corepressor complex^18^. The genetic defect leading to Smith-Magenis syndrome (SMS) is deletion of the *RAI1* (retinoic acid-induced 1) gene on 17p11.2. Also, Fragile X mutations block synaptic retinoic acid signaling, impairing neuronal homeostatic plasticity^19^. Also, mutations in the retinoic acid receptor beta gene, *RARB*, lead to intellectual disability with progressive motor impairment^20^. Another reported RDH14-interacting protein from the BioGRID dataset^15^ is the bone morphogenic protein/retinoic acid inducible neural-specific protein 2, BRINP2. Interestingly, copy number variants (CNV) at *BRINP2*’s orthologue, *BRINP1* (the *BRINP1*/*ASTN2* locus) have previously been linked with neurodevelopmental disorders, with a gain CNV in a patient with autism spectrum disorder (ASD), anxiety and learning disability, and a loss CNV in a patient with developmental delay and seizures, suggesting one or both of these genes having a role in neurodevelopmental disorders^21^. The *BRINP1*/*ASTN2* locus has also shown genetic linkage (LOD score = 4.11) with major psychiatric disorders in a single large pedigree from Spain (Pol-Fuster et al.^22^). However, GST pull-down using our WT and mutant RDH14-GST constructs was unable to confirm binding to BRINP2 (FAM5B) (see Supplementary Fig. S1).

In summary, owing to the intra-familial genetic heterogeneity in this family, additional analytic steps were required in order to make sense of the genetic architecture and likely disease-candidates. In addition to identifying a known disease-causing mutation in the *ADGRG1*/*GPR56* polymicrogyria gene in two of the four available affected members, the remaining two available affecteds both have a biallelic mutation in the retinol dehydrogenase gene, *RDH14*. MRI analysis from these two individuals at ages 20 months and 14 years suggests a progressive childhood-onset form of cerebellar atrophy, in addition to ID. While no supporting evidence yet exists from additional intellectual disability families or from animal studies, the relevance of *RDH14* to homeostasis of RA in the brain makes this a strong disease gene candidate.

## Materials and methods

### Recruitment

We identified a large consanguineous Pakistani family presenting with an apparently autosomal dominant form of hyperkeratosis across multiple branches, among which, in addition, there are four living and one deceased member with apparently autosomal recessive ID, including one male individual who presented with both ID and hyperkeratosis (see Fig. 1). Members of the family, who originate from the Bannu district in Khyber Pakhtunkhwa province in Pakistan, were recruited for a research study through Islamia College Peshawar, Pakistan, and Centre for Addiction and Mental Health (CAMH), Toronto, Canada. The study was approved by institutional ethics committees: Institutional Bioethical Committee (IBC), Islamia College Peshawar, under notification number 529/ORIC/ICP, and the Research Ethics Board, CAMH, Toronto. Informed written consent was obtained from all participants and/or their legal guardians, and the research was performed in accordance with relevant guidelines, and in accordance with the Declaration of Helsinki.

### Genetic/genomic analysis

After genomic DNA extraction using standard phenol/chloroform methods, Illumina CoreExome microarrays were run for individuals III-1, III-5, III-6, III-10 (affected), and III-2, III-7 and III-8 (unaffected) using an Illumina iScan^®^ microarray scanner (Illumina Inc, San Diego, USA). Microarray data was imported into Illumina Genome Suites, through which genotypes were extracted in PLINK format. Analysis of genotypes using the Homozygosity Mapper program (http://www.homozygositymapper.org^23^) was performed using default settings, firstly using all ID subjects classified as affected, and then, when no single HBD regions (> 1 Mb in length) was identified, the process was repeated reiteratively with different ID subjects reclassified as unaffecteds.

### Whole exome sequencing

Whole exome sequencing was performed for affected individuals III-1, III-5 and III-6. Whole exome paired-end sequencing was performed by Macrogen Inc. Republic of Korea Genome Sequencing Facility, using 2 μg of genomic DNA (260/280 > 1.7). Exome capture was performed using the Agilent 51 Mb SureSelect All Exon V4 kit (Agilent Technologies, Santa Clara, CA), and sequenced using the HiSeq 2000 platform (Illumina).

### Sequence analytic pipeline

Sequence reads were processed through an in-house custom analytic pipeline to map and call variants, as previously described^3^. Briefly, Illumina sequence reads were aligned with BWA-mem (v 0.7.13) using the hg19 genome reference^24^. After alignment GATK (v 3.2.2) was used for base recalibration, indel realignment, and the GATK Unified Genotyper was used for variant calling. Variants called were generated in the standard VCF version 4.1 and annotated with Annovar^25^. Once annotation was performed, filtration of variants was performed by computationally and manually extracting variants that were in HBD regions provided by the autozygosity mapping HomozygosityMapper. The next step was to filter variants based on sequencing quality/read depth, homozygosity of allele and mutation type. Prioritization was performed by scoring truncating mutations such as stop and frameshift loss of function mutations higher than missense mutations and inframe indels. Missense mutations were scored based on SIFT and Polyphen2, scores for prioritization, as well as meta-analysis algorithms Condel and CADD^26, 27^. Allele frequencies were then used to filter out variants that were too common in the population (> 1 in 10,000). All variants surviving filtration were checked against the gnomAD control database (https://gnomad.broadinstitute.org/).

### Sanger sequencing confirmation and segregation

Sanger sequencing was used to confirm the *ADGRG1* and *RDH14* mutations, and to check the segregation across the family. Sequencing was performed under service through Eurofins Genomics (Luxembourg). The PCR/sequencing primers used are listed in Supplementary Information.

### Cloning of *RDH14*, and site-directed mutagenesis

The *RDH14* coding sequence was amplified by PCR using attB1/2-flanked primers. The PCR template consisted of cDNA that was generated by reverse transcription of human brain RNA. Two sequential Gateway recombination reactions were utilized to generate the desired *RDH14* expression vectors. Briefly, the attB1/2-flanked *RDH14* amplicon was recombined into the donor vector pDONR221 using BP Clonase II, and then recombined into the desired Gateway expression vector using LR Clonase II. The Gateway expression vectors pDEST53 and pDEST15 (Thermo Fisher Scientific, Waltham, MA) were utilized to generate N-terminal GFP- and GST-tagged RDH14 recombinant proteins, respectively. To generate the mutant *GST-RDH14* variant, c.114_115insC (resulting in p.(Gly39Argfs*97) and identical to the mutation identified in the Pakistani family), site-directed mutagenesis using non-overlapping primers was employed. Briefly, PCR using the Q5 high-fidelity DNA polymerase and primers containing the desired mutation was used to replicate a linear version of the wildtype *RDH14*-pDEST15 vector, followed by phosphorylation of the 5′ ends of the amplicon using T4 polynucleotide kinase and subsequent ligation. Clones were screened for the desired mutation using Sanger sequencing (Supplementary Fig. S2). All primer sequences are listed in Supplementary Table S1.

### Immunocytochemistry

HEK293T cells [human embryonic kidney cells (ATCC CRL-3216TM)] were seeded onto 0.1 mg/mL poly-d-lysine-coated glass coverslips in a 24-well plate. When cells reached a confluence of approximately 50–70%, they were transiently transfected with 500 ng of the *RDH14*-pDEST53 vector using Lipofectamine 3000. 24 h post-transfection, cells were washed three times with ice-cold phosphate-buffered saline (PBS) and then fixed for 10 min at room temperature with 4% paraformaldehyde in 0.1 M PBS, pH 7.4, followed by three washes with PBS for 5 min. Cells were then permeabilized for 10 min at room temperature with 0.25% Triton X-100 in PBS. Following permeabilization, cells were washed three times with PBS supplemented with 0.1% Tween-20 (PBST) for 5 min, and then blocked for 1 h at room temperature in 1% bovine serum albumen (BSA) and 22.52 mg/mL glycine in PBST. Cells were then incubated with the primary anti-GFP antibody (ab290; Abcam, Cambridge, MA) in blocking solution (1:1000 dilution) overnight at 4 °C with gentle agitation in a humidified polystyrene container. The following day, cells were washed three times with PBST for 5 min, followed by incubation with the fluorophore-conjugated secondary antibody (A32731; Thermo Fisher Scientific, Waltham, MA) in blocking solution (1:1000) at room temperature for 2 h with gentle agitation and covered by aluminum foil (from this step, all subsequent steps were carried out under the cover of aluminum foil). Following incubation with the secondary antibody, cells were washed three times with PBST for 5 min, and then washed once with PBS, followed by incubation with DAPI (4′,6-diamidino-2-phenylindole; Roche-10236276001; MilliporeSigma; St Louis, MO) at a concentration of 1 μg/mL (dissolved in methanol) at room temperature for 5 min with gentle agitation. Cells were then washed twice with PBS for 5 min and mounted onto microscope slides using Dako mounting medium (Agilent Technologies, Santa Clara, CA).

### GST pull-down assays

GST-tagged *RDH14* and *RDH14*-Mut constructs were transformed in *E. coli* BL21 (D3) competent cells (C2527). GST, GST-RDH14 and GST-RDH14-Mut fusion proteins were prepared according to the Pierce™ GST Protein Interaction Pull-Down Kit manufacturer’s protocol (Cat# 21516, Pierce Biotech., Rockford, IL). Briefly, GST, GST-RDH14 and GST-RDH14-Mut fusion proteins were expressed in *E. coli* followed by isolation and treatment of *E. coli* with pull-down lysis buffer for 30 min on ice. Similarly, SK-N-SH cells (Human neuroblastoma (ATCC, Manassas, VA, USA)) were grown in Dulbecco’s Modified Eagle Medium (DMEM; (MilliporeSigma; St Louis, MO) containing 1% antibiotics and 10% FBS. SK-N-SH cell lysate was prepared using pull-down lysis buffer as described above and used as prey protein. GST fusion proteins were used as bait and equal amount were immobilized on an equilibrated glutathione agarose resin columns for 2 h at 4 °C. Columns were centrifuged, bait flow-through proteins were isolated and the columns were washed 5 times with wash solution (1:1 of TBS and pull-down buffer). Next, 800 µL of prey SK-N-SH cell lysate was added to the columns and incubated at 4 °C overnight. Columns were centrifuged, prey flow-through protein was isolated and then washed for a total of 5 times with wash solution. The resin-bound proteins were eluted in 10 mM glutathione elution buffer. The concentration of eluted proteins were measured and samples were prepared for western blot gel analysis.

For western blot analysis, protein was run on SDS-PAGE gels, transferred to PVDF nitrocellulose membrane and then incubated with 5% BSA blocking solution. The primary TMEM206 (PACC1), BRINP2 (FAM5B) and GST antibodies were attached and incubated overnight at 4 °C. The TMEM206/PACC1 protein was queried using anti-TMEM206 rabbit polyclonal antibody (1:1000 dilution; cat# TA342366; Origene Tech., Rockville, MD). The FAM5B/BRINP2 protein was queried using anti-FAM5B rabbit polyclonal antibody (1:500 dilution; cat# orb474203; Biorbyt Tech., St Louis, MO). GST was checked through hybridization with an anti-GST rabbit polyclonal antibody (1:1000 dilution; # 2622S; Cell Signaling Tech., Danvers, MA) (see Supplementary Fig. S3). The next day, membranes were washed with 1 × TBST solution (Tris Buffered Saline, with Tween 20, pH 8.0) followed by incubation with the HRP secondary antibodies for 1–2 h. Finally, membranes were sprayed with fluorescent solution and bands were visualized and obtained using the Gel Doc XR + System with Image Lab Software (Bio-Rad Laboratories, Hercules, CA).

### SiRNA knockdown of *RDH14* in SK-N-SH cells

The human neuroblastoma cell line SK-N-SH was cultured in DMEM medium supplemented with 10% FBS and 1% antibiotics at 37 °C in humidified air containing 5% CO_2_. When cells reached 80% confluency after ~ 24 h, the media was replaced with fresh serum and antibiotic free DMEM. The RDH14 protein expression was knocked down with the *RDH14* gene-silencing siRNA at a concentration of 75 pmol and 25 nMol per transfection for 40 h (Cat # AM16708, Thermo Fisher Scientific, Waltham, MA). Lipofectamine™ 3000 transfection reagent (Cat # L3000015, Invitrogen) was used to perform the transfection, followed by incubation at 37 °C and 5% CO_2._ The transfection reagents were also added to a ‘mock’ group without siRNA as control. After 40 h, cells were washed with ice cold PBS and collected for protein lysate preparation in RIPA buffer for immunoblotting. For western blot analysis, protein was run on SDS-PAGE gels, transferred to PVDF nitrocellulose membrane and then incubated with 5% BSA blocking solution. The primary TMEM206 (PACC1), BRINP2 (FAM5B), GST, RDH14, SOX2, MAP2, and β-actin antibodies were attached and incubated overnight at 4 °C. The membranes were then washed with 1 × TBST solution followed by incubation with the HRP secondary antibodies for 1–2 h. Finally, membranes were sprayed with fluorescent solution and bands were visualized and obtained using the Bio-Rad’s Gel Doc XR + System with Image Lab Software.

### RNA isolation and RT-qPCR

SK-N-SH cells intended for RT-qPCR were cultured in 6-well plates. 48 h after transient ‘mock’- or siRNA-transfection, cells were washed with PBS, and RNA was isolated using the NucleoSpin RNA kit (Machery-Nagel-Fisher Scientific, Dürel, Germany) according to the manufacturer’s instructions. The initial cell lysis was performed directly in the 6-well plate, and RNA was eluted in RNase-free water. RNA integrity was verified by agarose gel electrophoresis. RNA (1 μg) was reverse-transcribed into cDNA using the iScript cDNA synthesis kit (Bio-Rad). For qPCR, reactions were prepared using the PowerUp SYBR Green Master Mix (Thermo Fisher Scientific, Waltham, MA). For each reaction, cDNA samples were diluted either 10-, 100- or 1000-fold to ensure that amplification was occurring within the dynamic range for the given primer pair (primer sequences and annealing temperatures are provided). For each experimental condition (mock- or siRNA-transfected cells), four technical replicates were prepared for three biological replicates. Reactions were amplified in 384-well plates using the QuantStudio Real-Time PCR System software on the ViiA7 System instrument (Thermo Fisher Scientific, Waltham, MA) with the ΔΔC_t_ experimental setting and standard amplification properties. Each reaction was subjected to melt-curve analysis to ensure the presence of only one amplicon, and a non-template control reaction was amplified in parallel for each primer pair. For each biological replicate, C_t_ values from two reference genes, *GAPDH* and *18S*, were averaged (C_t_-reference) and used for normalization. For normalization of experimental genes for each biological replicate, C_t_ values from the four technical replicates were averaged, and the C_t_-reference value was subtracted (ΔC_t_). In order to evaluate gene expression relative to the mock-transfection, the geometric mean of the ΔC_t_ values from only the three mock-transfection biological replicates was calculated, and subtracted from the ΔC_t_ values from each biological replicate across both mock- and siRNA-transfected conditions (ΔΔC_t_). Lastly, 2^−ΔΔCt^ was calculated for each biological replicate.

## Supplementary Information

Supplementary Information.

## Data Availability

Information on the variant reported here has been submitted to the ClinVar database housed by NCBI: ClinVar accession #SCV001167015. The microarray and WES data that support the findings of this study are available from the corresponding author upon reasonable request.
