# Supplementary material for: Biallelic inheritance in a single Pakistani family with intellectual disability implicates new candidate gene RDH14
Source: Sci Rep. 2021 Nov 30;11:23113. doi: 10.1038/s41598-021-02599-z (PMC8632963; doi:10.1038/s41598-021-02599-z)

# Complex biallelic inheritance in a single Pakistani family with intellectual disability implicates new candidate gene *RDH14*

Stephen F Pastore, Tahir Muhammad, Ricardo Harripaul, Rebecca Lau, Muhammad Tariq Masood Khan, Muhammad Ismail Khan, Omar Islam, Changsoo Kang, Muhammad Ayub, Musharraf Jelani, John B Vincent

**Supplementary Information**

**Contents:**

**Clinical Information**

**Supplementary Figure S1**

**Supplementary Table S1**

**Supplementary Figure S2**

**Supplementary Figure S3**

**Uncropped western blots**

The individuals from the Bannu family identified with the homozygous truncating mutation in *RDH14* (and not the homozygous truncating mutation in *GPR56*/*ADGRG1* polymicrogyria gene, which is present in several family members) were a 16-year-old male (III:1) and a 6-year-old female (III-10) with developmental delay. They both had profound intellectual disability. They were both born at full term, after uneventful pregnancies. The parents had noticed their low birth weight soon after birth. Psychomotor developmental delay was noticed at the age of 6 months. The patients were examined upon recruitment into the study; the brother scored at level V on the Gross Motor Function Classification System^1^ (GMFC) scale, while the sister scored at GMFC level II. The motor weakness remained static, and did not appear to progress or regress with time. Weight and height were below average for both, while BMI was found to be in normal range. There were no obvious anatomical anomalies in either subject. They both had aphasia (uttering incomprehensible sounds) and dyskinesia. Higher traits, like self-actualization and esteem were absent, and love/belonging and safety instincts were minimally present. Both subjects were urine and fecal incontinent, and needed assistance feeding. Ophthalmologic examination was normal. The subjects demonstrated heterogeneity in play behavior: the girl exhibited a calm behavior while the boy manifested aggression.

By the age of ~13, the male subject (III-1) was completely bed-bound, and died while still a teenager of unknown medical causes. The female (III-10) was able to walk, although with abnormal gait and poor balance. She is reported by the family to have since died falling down stairs.

1. Paulson A, Vargus-Adams J. Overview of Four Functional Classification Systems Commonly Used in Cerebral Palsy. Children (Basel). 2017 Apr 24;4(4):30. doi: 10.3390/children4040030. PMID: 28441773; PMCID: PMC5406689.

**Supplementary Figure S1:** **Western blotting for GST pull-down of FAM5B/BRINP2 protein** **from SK-N-SH cell lysate using wild-type (WT) versus mutant RDH14-GST constructs.** **A.** Pull-down using RDH14-GST, RDH14-Mut-GST, and GST constructs, and detection of FAM5B/BRINP2 protein using anti-FAM5B rabbit polyclonal antibody (1:500 dilution; cat# orb474203; Biorbyt Tech., St Louis, MO). Input to lane 1 was SK-N-SH cell lysate (as a positive control). Lanes 2 and 3 show extracted protein for the RDH14-GST constructs, WT and mutant, respectively (as negative controls). In lanes 4, 5, and 6, the GST pull-down eluted protein, for WT and mutant RDH14-GST, and GST alone (as negative control), respectively (i.e. bait protein against prey input). **B.** Flow-through from lanes 2-6 above was run and probed with the anti-FAM5B antibody.


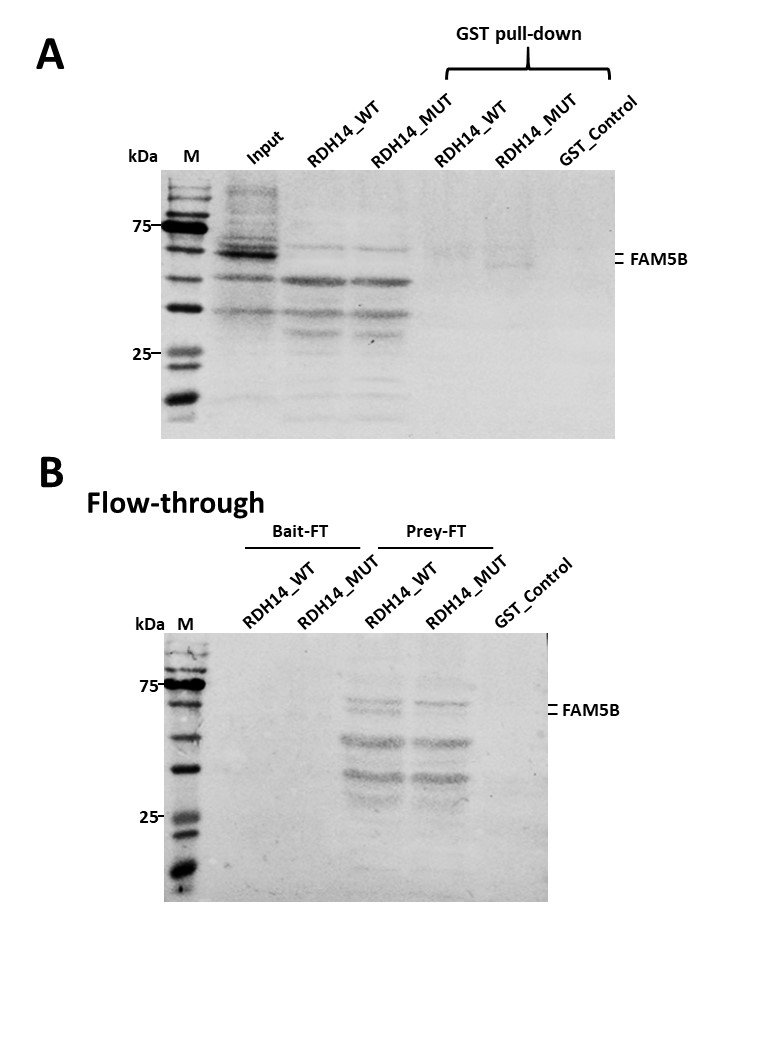


**Supplementary Table S1: Primers. A. Primers for validation and segregation of ADGRG1 (GPR56) and RDH14 variants. B. Primers for cloning the RDH14 cDNA and for site directed mutagenesis.** These primers generated a mutation identical to that discovered in the Bannu family. The primers were designed to anneal back-to-back in order to replicate the entire vector, with the mutant forward primer containing an extra cytosine at the 5’ end representing the insertion. The inserted cytosine base is underlined in the mutagenesis forward primer. **C. Primers for qRT-PCR of genes transcribed in SK-N-SH cells.**

| **Primer** | **Sequence (5’ to 3’)** |
| --- | --- |
| 1. **ADGRG1 and RDH14 validation and segregation** | |
| RDH14_F | CTGGAGCATTTCCTGGCAG |
| RDH14_R | CGTACAGGTGGTCTCTTGG |
| GPR56ex5F | CTCCTCTGTCTGACCCTAC |
| GPR56ex5R | TTCAGGGAGTGCTCACAGC |
| 1. **RDH14 cDNA cloning and site-directed mutagenesis** | |
| RDH14_attB1_F | GGGGACAAGTTTGTACAAAAAAGCAGGCTCCATGGCAGTGGCCACTGCGGC |
| RDH14_attB2_R | GGGGACCACTTTGTACAAGAAAGCTGGGTCTATTTTAGCAGGCCAACCATCACTTCACTG |
| RDH14_mut_F | CCCCCGGCCTCATGCACGG |
| RDH14_mut_R | TCCCCGCCTCTGCGCAGC |
| 1. **qRT-PCR for siRNA RDH14 knockdown of SK-N-SH cells** | |
| *GAPDH* | TTCTTTTGCGTCGCCAGCCGA |
|  | GTGACCAGGCGCCCAATACGA |
| *18S* | GTGGTGTTGAGGAAAGCAGACATTG |
|  | CATCCTTCTGTCTGTTCAAGAACCAGT |
| *OCT4* | CCCCAGGGCCCCATTTTGGTACC |
|  | ACCTCAGTTTGAATGCATGGGAGAGC |
| *SOX2* | TTCACATGTCCCAGCACTACCAGA |
|  | TCACATGTGTGAGAGGGGCAGTGTGC |
| *NESTIN* | TCAAGATGTCCCTCAGCCTGGA |
|  | AAGCTGAGGGAAGTCTTGGAGC |
| *KI67* | GAAAGAGTGGCAACCTGCCTTC |
|  | GCACCAAGTTTTACTACATCTGCC |
| *NEUROD1* | GGTGCCTTGCTATTCTAAGACGC |
|  | GCAAAGCGTCTGAACGAAGGAG |
| *RAR-A* | CATCCCCAGCCACCATTGAGAC |
|  | GACAGACAAAGCAAGGCTTGTAGATG |

**Supplementary Figure S2: Sanger confirmation of site-directed mutagenesis.** The red dashed box indicating inserted cytosine.


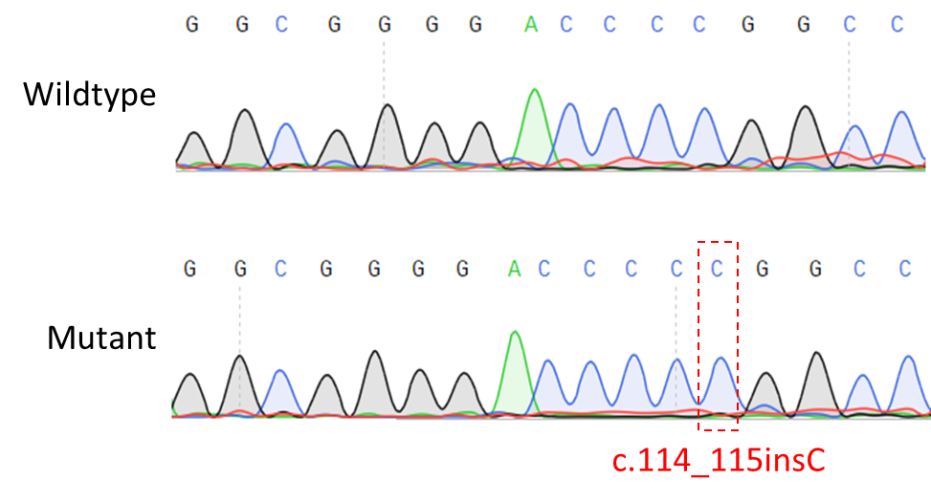


**Supplementary Figure S3:** Representative image of western hybridization using the GST-fused RDH14, RDH14-Mut, and GST control proteins used in the GST pull-down assay, using anti-GST rabbit polyclonal antibody (1:1000 dilution; # 2622S; Cell Signaling Technologies, Danvers, MA).


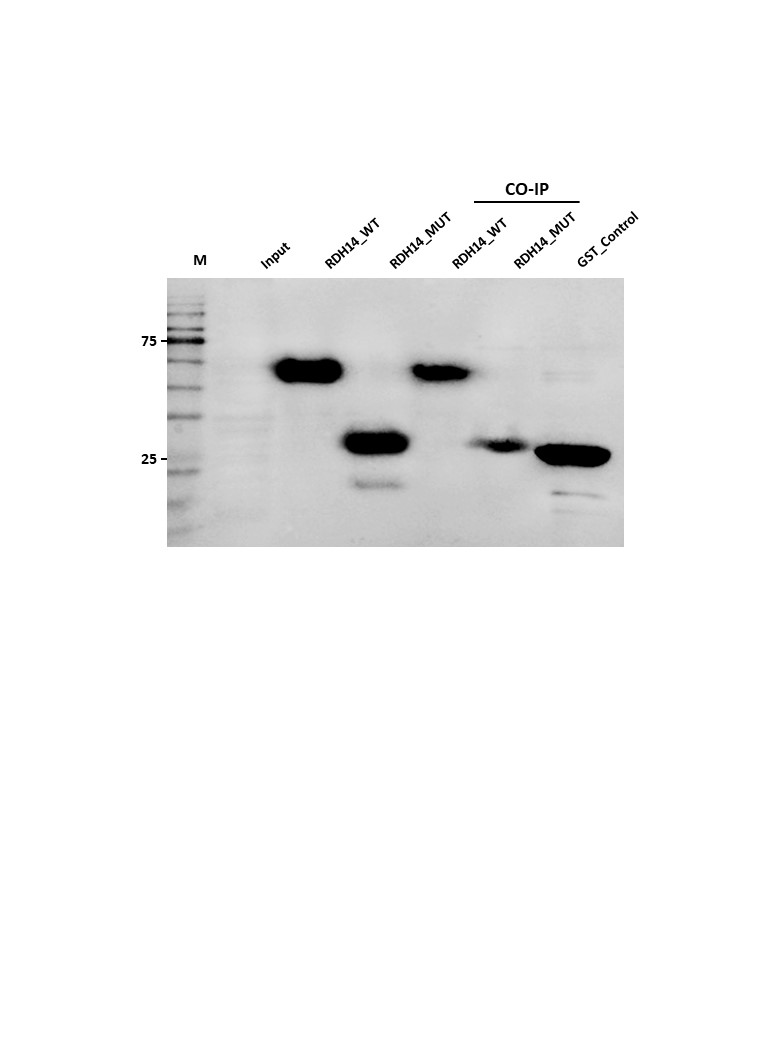


**Supplementary Figure S4: Uncropped blots**

**Main text: Figure 3A Main text: Figure 3B**


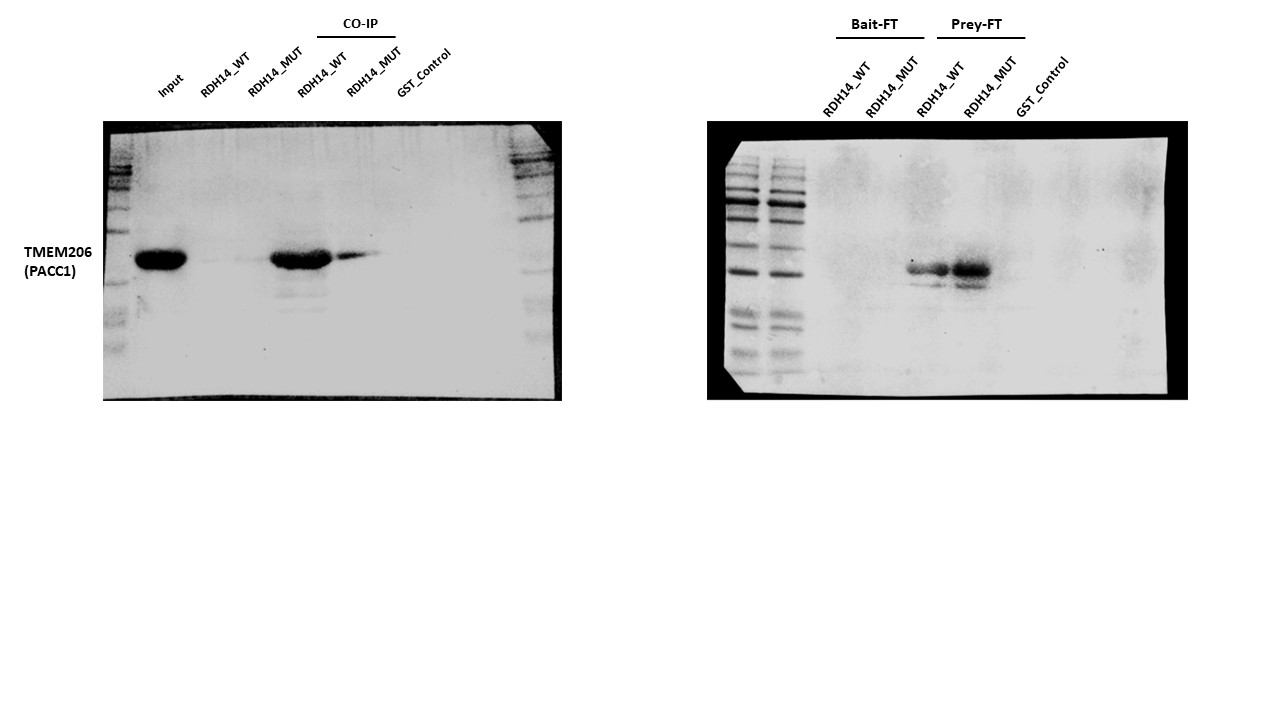


**Main text: Figure 4A Main text: Figure 4A**

**
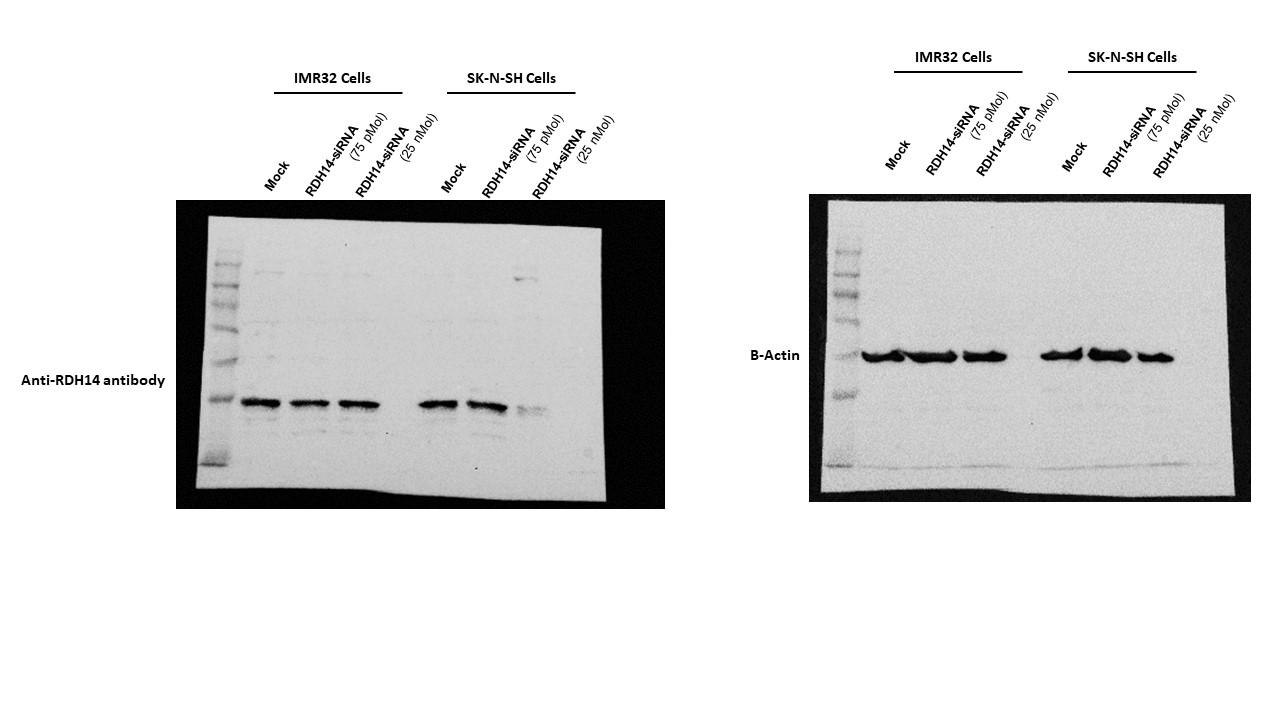
**

**Main text: Figure 4C**

**
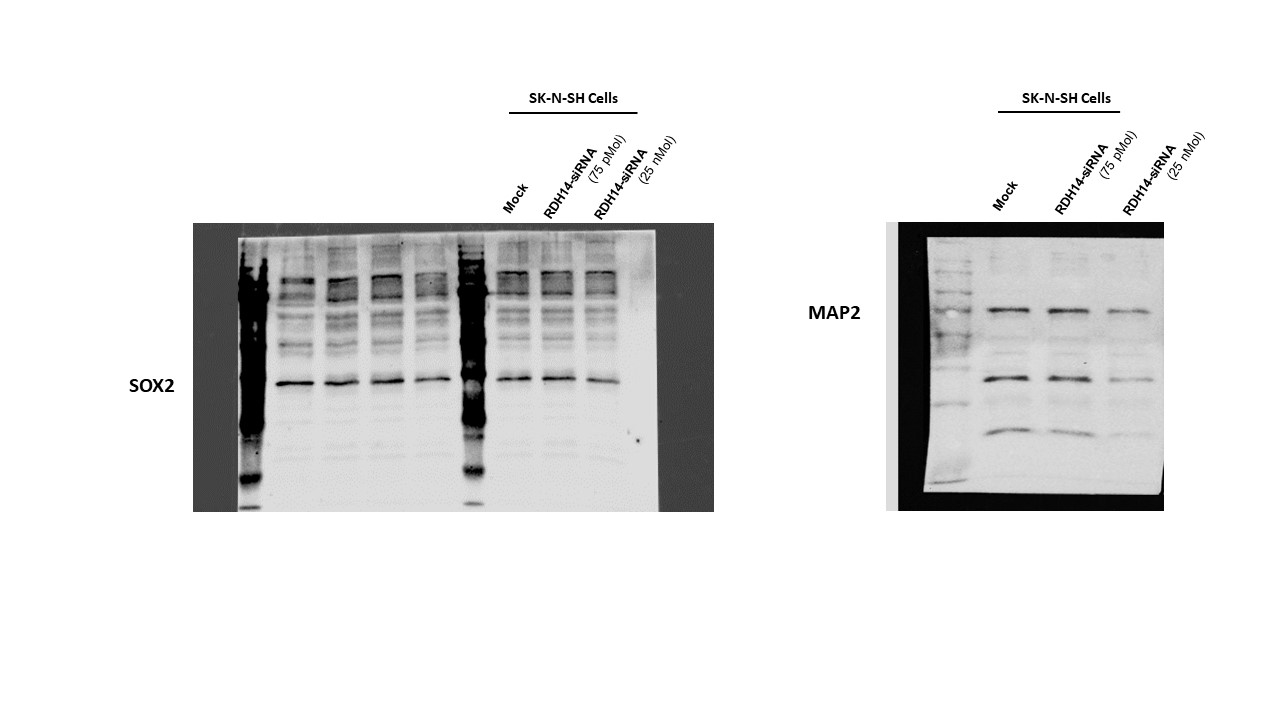
**

**Figure 4D**

**
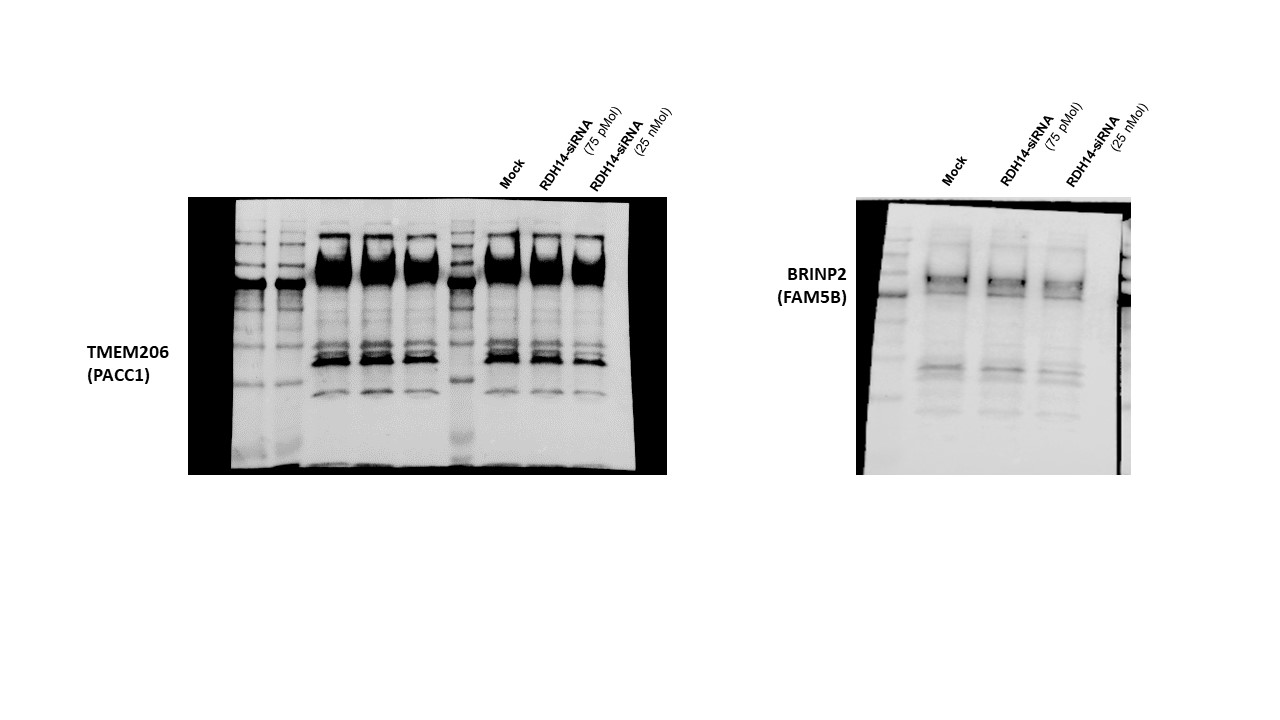
**

**Figure 4C & D: β-actin**

**
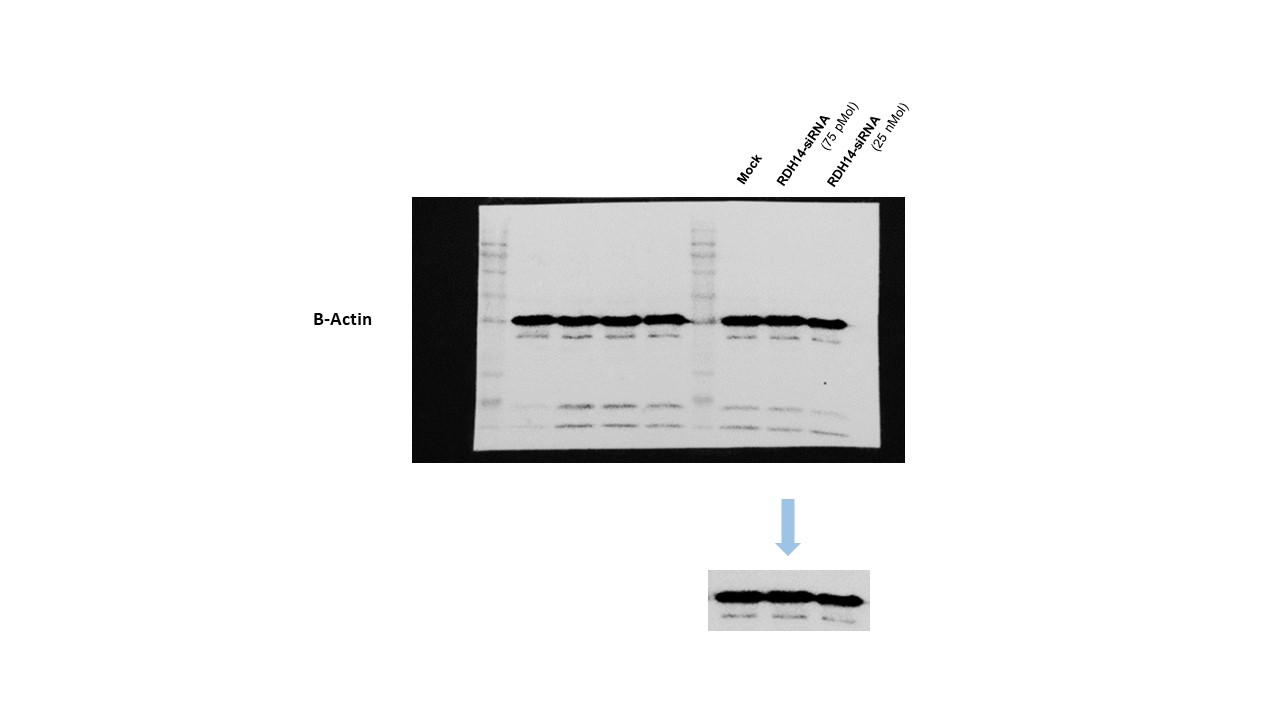
**

**Supplementary Information: Figure S1A Figure S1B**


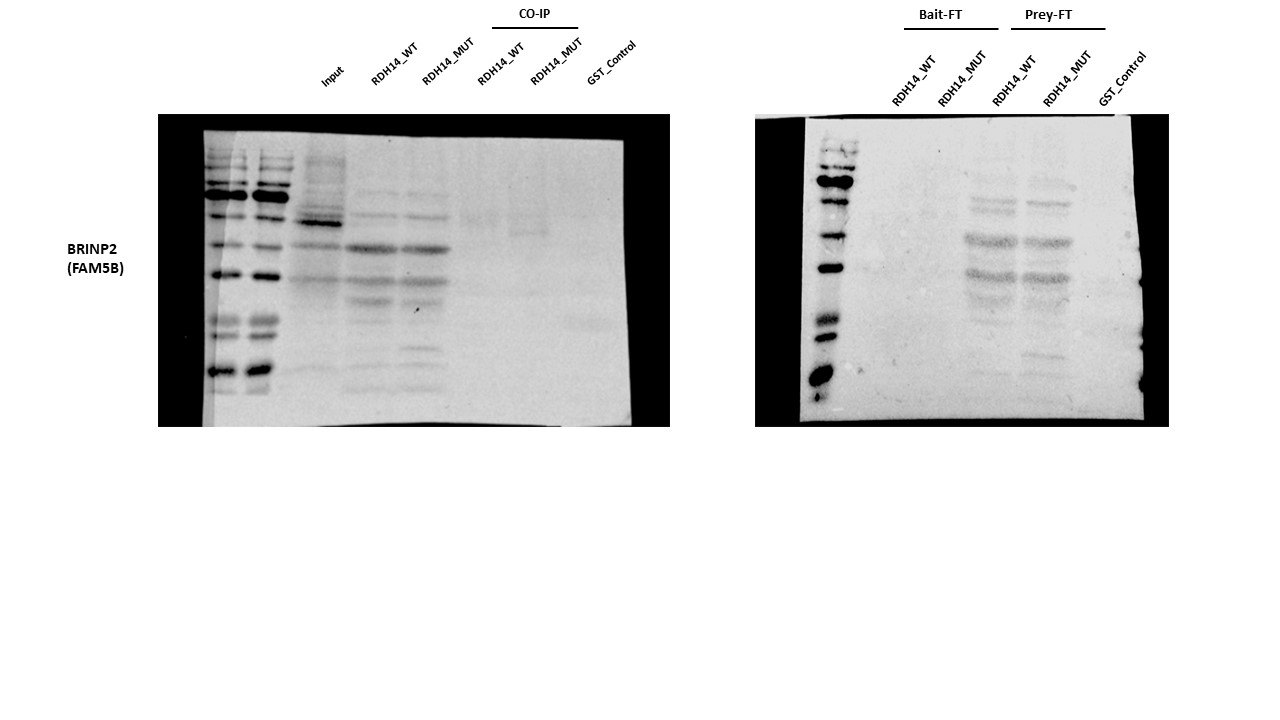


**Supplementary Information: Figure S3**


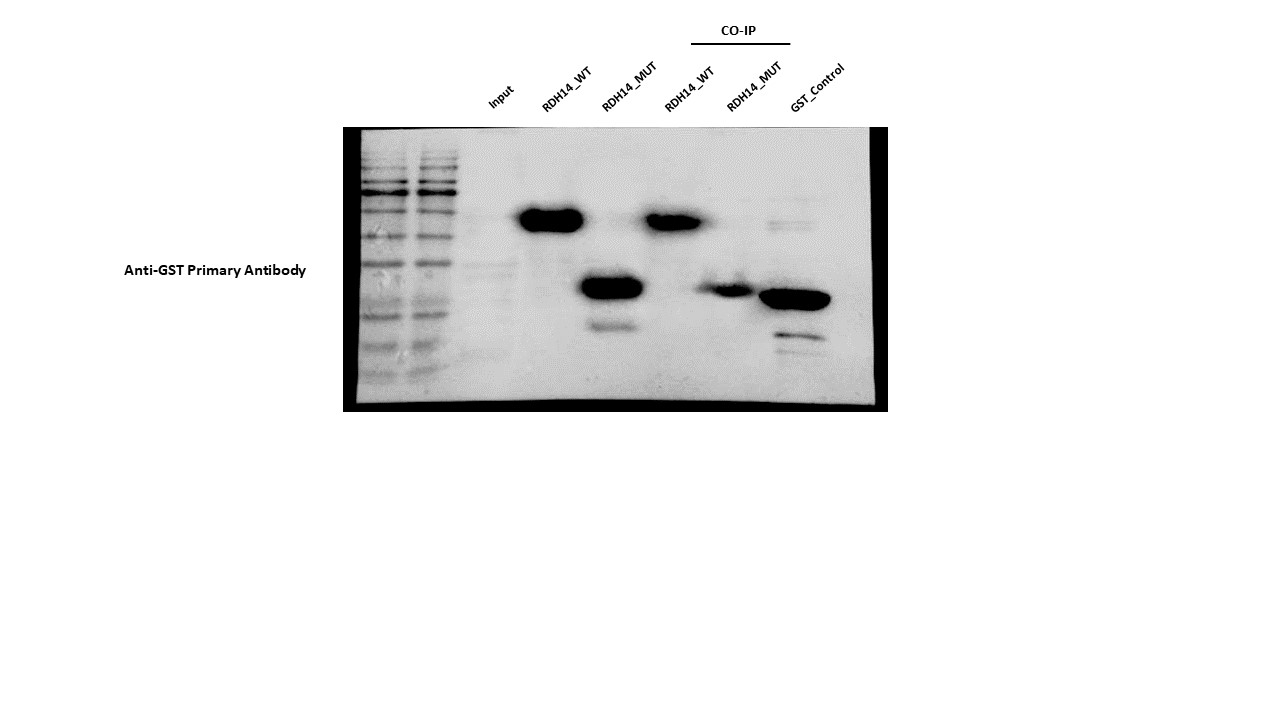

Supplement: Supplementary file 1 — Supplementary Information. [file 41598_2021_2599_MOESM1_ESM.docx]
